# Wood Polymer Composites Based on the Recycled Polyethylene Blends from Municipal Waste and Ethiopian Indigenous Bamboo (*Oxytenanthera abyssinica*) Fibrous Particles Through Chemical Coupling Crosslinking

**DOI:** 10.3390/polym16212982

**Published:** 2024-10-24

**Authors:** Keresa Defa Ayana, Abubeker Yimam Ali, Chang-Sik Ha

**Affiliations:** 1 Process Engineering Stream, School of Chemical and Bio Engineering, Addis Ababa Institute of Technology, Addis Ababa University, King George VI St., Addis Ababa 1000, Ethiopia; 2 Department of Polymer Science and Engineering, School of Chemical Engineering, Pusan National University, Busan 46241, Republic of Korea

**Keywords:** wood–plastic composite, interfacial compatibility, mechanical properties, bamboo fibrous particles, cascading thermoplastic waste

## Abstract

Valorization of potential thermoplastic waste is an effective strategy to address resource scarcity and reduce valuable thermoplastic waste. In this study, new ecofriendly biomass-derived wood polymer composites (WPCs) were produced from three different types of recycled polyethylene (PE) municipal waste, namely linear low-density polyethylene (LLDPE), medium-density polyethylene (MDPE), or high-density polyethylene (HDPE), and their blend with equal composition (33/33/33 by wt.%). Bamboo particle reinforcement derived from indigenous Ethiopian lowland bamboo (LLB), which had never been utilized before in a WPC formulation, was used as the dispersed phase. Before utilization, recycled LLDPE, MDPE, and HDPE were carefully characterized to determine their chemical compositions, residual metals, polycyclic aromatic hydrocarbons, and thermal properties. Similarly, the fundamental mechanical properties of the WPCs, such as tensile strength, modulus of elasticity, flexural strength, modulus of rupture, and unnotched impact strength, were evaluated. Finally, the thermal stability and interphase coupling efficiency of maleic-anhydride-grafted polypropylene (MAPP) were carefully investigated. WPCs formulated by melt-blending either of the recycled PEs or the blend of recycled PE with bamboo particles showed significant improvement due to MAPP enhancing interfacial adhesion and thermally induced crosslinking, despite inherent immiscibility. These results were confirmed using Fourier transform infrared spectroscopy, scanning electron microscopy, and thermogravimetric analysis. The formulated WPCs may promote PE waste cascading valorization, offering sustainable alternatives and maximizing LLB utilization. Furthermore, comparison with well-known standards for polyolefin-based WPCs indicated that the prepared WPCs can be used as alternative sustainable building materials and related applications.

## 1. Introduction

The growing demand for resources and environmental sustainability have emerged as a paramount concern to search for sustainable materials and efficient utilization of resources [1]. In particular, the use of fossil-fuel-based nonbiodegradable plastics has steadily increased, contributing to solid plastic waste (SPW) [2,3,4]. Polyethylenes (PEs) are highly accumulated as SPW (50–70%) [5], with infiltration in different parts of the environment. Such difficult situations and resource wastage demand sustainable utilization to minimize the PE waste fractions [5,6,7]. Till now, a few methods have been utilized, including thermomechanical recycling [8], incineration and common landfills [9], pyrolysis of multipolymer systems, and depolymerization techniques [10]. Alternative technologies have also been attempted for valorizing thermoplastic waste with eco-friendly biomass materials and reintegrating them into the product life cycle before incineration or landfilling [11].

Wood polymer composites (WPCs) have emerged as promising materials for post-consumer PE plastic waste valorizations with sustainable natural fibers [2]. Moreover, unlike mineral fibers, plant fibers used in wood–plastic composites (WPCs) are eco-friendly, widely available, and significantly cheaper than synthetic fibers like carbon, glass, and aramid. While mineral fiber-based composites undeniably offer superior mechanical properties, they are costly, environmentally unfriendly, and unsustainable [1,6]. Furthermore, WPCs formulated from secondary resources and bio-based natural fibers are sustainable and potentially used as the intermediate cascade chains for underutilized lignocellulosic biomass and thermoplastic waste [12,13]. Additionally, it can address issues of energy-intensive conventional building materials, such as cement, which releases one ton of CO_2_ per ton of production, owing to energy-intensive calcination reactions [14]. Similarly, structural materials, such as Fe and steel, also contribute to approximately 11% of industrial CO_2_ emissions [3]. Although these construction materials cannot be easily replaced, WPCs can be used in various diverse applications, from buildings and construction (e.g., decking and cladding) to infrastructure and transportation (e.g., automotive parts), and various outdoor products [15,16]. This combines the desirable properties of lignocellulosic fibrous fillers and/or reinforcements with the processability of thermoplastic polymers, making it suitable for high-volume production using standard plastic-manufacturing techniques [17].

Lignocellulose fibers contain complex molecules, such as cellulose, hemicelluloses, lignin, and pectin [3], which are physiochemically different from thermoplastic polymers. These complex molecules are rich in polar hydroxyl (–OH) and carbonyl groups which weakly combine with nonpolar hydrophobic matrix polymers, resulting in a weak interphase [15,18]. Hence, improving the interfacial compatibility between the two distinct WPC phases is a critical aspect of WPC formulations. These techniques mostly include the use of coupling agents, silanization, controlled free-radical grafting, and grafting polymerization [19,20,21,22]. Chemical coupling and crosslinking agents have garnered significant commercial presence over traditional solution-based treatments [23]. They efficiently form bonds through various mechanisms, such as diffusion, electrostatic attraction, chemical reactions, and secondary bonds, such as hydrogen bonds and van der Waals forces [23]. In WPC formulations, chemical coupling and crosslinking agents can be applied through in situ reactive compatibilization and multimonomer graft copolymerization [24,25] without generating secondary waste, unlike solution-based processes, which make it difficult to maintain optimal levels [26].

Recently, various studies have been performed using polymeric thermoplastic matrices and different lignocellulosic fibers for WPC production. Polyolefins, such as linear low-density PE (LLDPE) [27], HDPE [28,29], PP [18,30,31,32], PVC [33,34], and acrylonitrile-butadiene-styrene [35,36], have been used as matrix polymers for WPCs [3,37]. However, no studies have utilized indigenous Ethiopian lowland bamboo (*Oxytenanthera abyssinica, hereafter abbreviated as “LLB”*) as the dispersed phase in WPCs combined with three well-characterized and well-known PE waste poly blends as the polymeric matrix.

The LLB has solid cross-sections, grows in Ethiopia lowlands, and covers approximately an area of one million hectares [38,39]. It is a socio-economically and ecologically valuable plant indigenous to Ethiopia and endemic to tropical Africa [40]. Thus, the main objective of this study is to explore the combined use of three potential recycled PE waste fractions, namely recycled LLDPE (rLLDPE), recycled MDPE (rMDPE), and recycled HDPE (rHDPE) as polymeric matrices, along with the untapped potential of indigenous LLB as reinforcement particles. We utilized its particles without fiber extraction, which is energy-consuming and leaves almost no carbon footprint. Additionally, we explore how immiscibility between phases can be improved with maleic-anhydride-grafted polypropylene (MAPP), which has wider commercial footprint. The WPC formulations consist of either of the three different recycled polyethylene (rLLDPE, MDPE, or rHDPE) or their equal-composition blend (33/33/33 by wt.%) as the matrix and short fibrous LLB particles of wider distribution, ranging from 200 to 600 µm, as the dispersed phase aiming to investigate their wide-range reinforcement. The comprehensive mechanical performances of the composites, including the coupling efficiency of the MAPP compatibilizer, were carefully investigated. Finally, the WPCs that satisfy the minimum WPC standards were further evaluated for thermal degradation and stability and characterized with FTIR and scanning electron microscopy (SEM) for interphase compatibility. The formulations involved mixing and compounding various compositions of the polymer matrix and LLB particles using a pilot-scale Haake mixer configured with twin-screw extruders, followed by hot-press consolidation to create a WPC board prototype.

## 2. Materials and Methods

### 2.1. Post-Consumer Thermoplastic and Low-Land Bamboo Fibrous Particles

Waste fractionated from recycled post-consumer PE-related materials was collected from bulky urban waste streams in Addis Ababa and classified as rLLDPE, rMDPE, and rHDPE, first based on their resin codes and later by characteristics melting behavior and densities including their crystallinity fractions. They were further treated to obtain plastic grades of higher quality, involving size reduction, washing, regrinding, and pelletization using a counter-rotating extruder. The densities of the recycled polymers and coupling agents were determined according to ASTM D792-20 [41], and the melt flow indexes (MFIs) were evaluated using ISO 1133 [42], as shown in Table 1. PP grafted with 8–10% maleic anhydride (MA) (Mw~9100, GPC) from Sigma-Aldrich Korea (Seoul, Republic of Korea) was used as the coupling agent. Matured LLB culms were harvested from Arjo Gudatu (9°4′52″ N, 36°37′30″ E) district of the Oromia regional provinces in Ethiopia. After removing the topmost part, the sample was cut into small strips, air-dried, and pulverized using a micronized cross-beater mill (model SM 2000, Retsch GmbH, Haan, Germany). Subsequently, the LLB particles were sieved to the target size of 200–600 µm with wide distributions ranges. Finally, they were dried in a vacuum oven at 90 °C until the moisture content dropped below 2% and were used directly for the WPC formulations.

### 2.2. Free Sugar Content, Cellulose, Hemicellulose, and Lignin Analysis of LLB

The chemical characterization of the LLB was performed according to Lorenz et al. [43], using two-stage acid hydrolysis and high-performance anion exchange chromatography coupled with ultraviolet–visible spectrophotometry (HPAEC–UV/VIS). The entire process is summarized as follows. The milled and dried biomass was hydrolyzed by adding 100 mg of each sample to 1 mL 72% H_2_SO_4_. The suspensions were conditioned to 30 °C for exactly 60 min before being hydrolyzed. After further dilution to 2.5% H_2_SO_4_, the samples were treated in an autoclave for 30 min at 120 °C, and the hydrolysis residue was filtered, washed, dried, and weighed. The filtered solutions were analyzed using the chromatographic borate HPAEC analysis using Dionex Ultimate 3000 (Dionex Corporation, CA, USA), anion exchange resin MCL GelCA08F (Mitsubishi Chemical Corporation, Tokyo, Japan), and a 5 mm × 120 mm Omnifit column, (Diba Industries, Inc., Danbury, CT, USA) packed at 65 °C for chromatographic separation. The mobile phase of two potassium tetraborate/boric acid-buffers in water with 0.3 M solution of pH 8.6 (C) and 0.9 M solution of pH 9.5 (D) were used. Separation was performed at 65 °C with a flow rate of 0.7 mL/min, and the elution program was started with 90% C and 10% D solutions, which were changed to 10% C and 90% D solutions after 35 min. This rate was kept constant for 8 min and changed back to 90% C and 10% D in 7 min. Before detection, post-column derivatization by Cu–bicinchoninate (0.35 mL/min) was applied at 105 °C in a Teflon^®^ coil of 30 m length and 0.3 mm diameter according to the literature [43]. Absorbance was measured at 560 nm using UV–vis detection. Finally, characterization was performed with the following conditions: equal fraction of cellulose from glucose, total sum of xylose, mannose, galactose, arabinose, and rhamnose for hemicellulose, and total sum of the hydrolysis residue and acid-soluble lignin for lignin.

### 2.3. Chemical Structure and Thermal Properties of the Recycled Polymeric Matrix and Its WPCs

The chemical structures, melting characteristics, and thermal properties of post-consumer rLLDPE, rMDPE, and rHDPE were analyzed. FTIR analysis of the polymeric matrix films initially prepared by hot-pressing and WPC samples was performed using KBr matrix-bound pellets under hydraulic pressure (60 N/m^2^). An Agilent (Model Cary 640, Santa Clara, CA, USA) FTIR spectrometer was used to scan the samples from 4000 to 400 cm^−1^ at 64 scanning rate with an optical resolution of 4 cm^−1^. The neat reference LDPE spectra were used for comparison. For the thermogravimetric analysis (TGA), 10 mg samples of both recycled plastics matrices and WPCs were analyzed using a Q50 TGA instrument (New Castle, DE, USA) in a nitrogen atmosphere (flow rate: 60 mL/min) from 30 to 650 °C at 10 °C/min. First-derivative thermogravimetric analysis (DTGA) was used to analyze the temperature-dependent transition peaks. DSC was performed using 6 mg of recycled PE on a TGA instrument (STA Q600, New Castle, DE, USA) in an inert nitrogen environment (flow rate: 60 mL/min). The heating and cooling cycles were implemented at rates of 10 °C/min in the range of 25–350 °C to determine the crystallization temperature (T_c_), melting temperature (T_m_), melting enthalpy (ΔH_m_), and crystallization enthalpy of the recycled polymeric matrices. Finally, the degree of crystallinity (X_C_) of the recycled PE was calculated based on the ratio of the absorbed enthalpy during the heating process to that of the highly crystallized pure PE (293 J/g) [44].

### 2.4. Analyses of Contents of Residual Metal Ions and Polycyclic Aromatic Hydrocarbons (PAHs) in the Recycled PEs

The PAHs were determined following the test procedures and guidelines used in a previous work [45] which can be summarized as follows: Approximately 500 mg rLLDPE, rMDPE, and rHDPE samples were extracted with 20 mL toluene for 1 h at 60 °C in a 200 W ultrasonic bath of surface area of 706 W/cm^2^. An aliquot of the extract was cooled to room temperature (25 °C) and purified using column chromatography. The PAHs were determined using gas chromatography coupled with mass spectrometry, employing the selective-ion-monitoring method. Samples (1 µL) were injected using pulse splitless with the separation involved in the HT8 column (25 m long, 0.22 mm internal diameter, 0.25 µm film thickness). The injector temperature was 280 °C, and the transfer-line temperature was 260 °C. The initial and final temperatures were 50 and 320 °C, with the initial and final times of 2 and 8 min, respectively, at a heating rate of 11 °C/min. Similarly, the use of recovered thermoplastic waste should follow a specific threshold for residual heavy metals impurities to ensure end-user safety. Furthermore, large residual metal ions can catalyze the thermo-oxidation or photo-oxidation of matrix polymers, leading to unwanted polymerization and emission of volatile organic compounds, resulting in uncontrolled porosity in WPC materials and affecting their durability [17]. The residual elements, specifically heavy metals impurities, in rLLDPE, rMDPE, and rHDPE were assessed according to the analytical parameters adhering to TOXEL standards developed by DSM Resolve and PANalytical which includes certified standards for regulated elements, such as Cr, Cd, Hg, Pb, As, Ni, Cu, Zn, Ba, and Br [46,47].

### 2.5. Formulation of WPCs

WPC preparation involved the compounding and consolidation of two compositions of LLB particle reinforcements (40 and 60 wt.%). Formulations involving a total of 20 samples, including unreinforced polymer matrices of rLLDPE, rMDPE, rHDPE, and their blend (EM) with equal composition (33/33/33 by wt.%), are shown in Table 2. The first letter of the polymer matrix is used with the subscript number to indicate their compositions in the matrix composites, and LB represents the LLB particle reinforcement. During compounding, the polymeric matrix was added to the preheated mixing chamber (HAAKE Reomix) and melted for approximately 5 min at 150, 160, 170, and 165 °C for rLLDPE, rMDPE, rHDPE, and rEM, respectively. Subsequently, the dried LLB particles and MAPP were added and homogeneously compounded for an additional 10 min until constant torque and viscosity were achieved. Finally, the dried WPC granules were further reduced using a cutting mill (model SM 2000, Retsch GmbH, Haan, Germany) with a mesh size of 8 mm. The consolidation of the WPC boards was performed in a computer-controlled hot-compression press with a steel mold frame of dimensions of 180 mm × 200 mm × 4 mm. This process involved cycles of low-pressure melting for 1 min, compression at 20 bar for approximately 8 min, continuous pressing by increasing the pressure to 60 bar for the next 2 min, and pressing at 100 bar for an additional 2 min. While maintaining a constant pressure at 100 bar, pressing continued cooling with an external water coolant flow until the temperature decreased to less than 80 °C for an additional one minute. Finally, the WPC board prototype was removed from the frame.

### 2.6. Sample Preparation for Mechanical Tests

**Figure 1:**
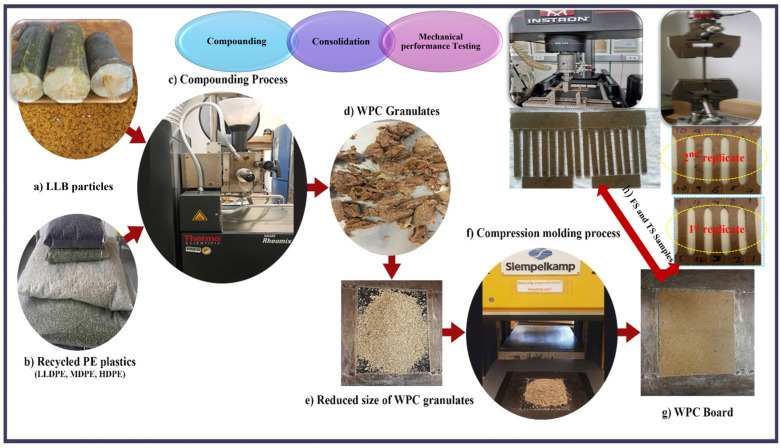
Compounding, consolidation, and mechanical testing of the WPCs.

One of the advantages of WPCs is their machinability. The dumbbell-shaped tensile test specimens were cut using a precise cutting machine, which was guided by software(cncGraFPro-AL640PROFI2-1705) according to the selected standard. For the impact and flexural strength tests, we adjusted the table saw based on the applicable standards, and the specimen samples were cut. Five independent specimens from each replicate were used to evaluate the mechanical properties. The tensile strength (TS) was evaluated using 10 dumbbell-shaped test specimens with dimensions of (170 mm × 10 mm × 4 mm) from the two replicates, following ISO 527-1 [48] requirements. A universal testing machine (ZwickRoell GmbH & Co. KG, Ulm, Germany) was used to test the mechanical properties. The load cell and crosshead speeds were 20 kN and 1 mm/min, respectively. The tensile modulus (TM) was determined in the strain range of 0.05–0.25%. The flexural strength (FS) and modulus of rupture (FM) were measured for specimens with dimensions of 80 mm × 10 mm × 4 mm according to the test compliance of ISO 178-1 [49] with a span length of 60 mm and crosshead speed of 1 mm/min. Unnotched impact strength (UIS) was measured following ISO 179-2 [50] set at 80 mm using a Charpy impact tester (Zwick-Roell HIT5.5P, GmbH & Co. KG, Ulm, Germany) for approximately 12 samples from the two replicates with similar dimensions as those of the FS (Figure 1).

### 2.7. Scanning Electron Microscope (SEM) Observation

SEM micrographs of the fractured surfaces of representative WPC samples from the IS tests (1 cm × 1 cm × 1 cm) were examined using a QUANTA 200 scanning electron microscope of (Thermo Fisher Scientific, Hillsboro, OR, USA) to obtain the indirect information on the interfacial adhesion between the matrix and particles. The surfaces were sputter-coated with a 2 nm Pt layer to enhance conductivity, and observations were conducted at an accelerated electron beam voltage of 10.0 kV.

## 3. Results and Discussion

### 3.1. Characterization of Ethiopian Low-Land Bamboo (Oxytenanthera abyssinica)

The amounts of free sugar and corresponding polysaccharides of xylose, glucose mannose, galactose, arabinose, and rhamnose in indigenous Ethiopian LLB were 12.05%, 57.16%, 0.62%, 0.39%, 1.16%, and 0.07%, respectively. Accordingly, the cellulose, hemicellulose, and lignin contents were 57.16%, 14.29%, and 25.94%, respectively. LLB contains a higher cellulose content than other bamboo fibers of different species, with lower hemicellulose content and similar lignin content [26]. Additionally, the amounts are comparable to widely utilized lignocellulosic fibers, such as hemp, jute, oak, and pine fibers, which is potential to replace conventional fibrous fillers and/or reinforcements [37].

### 3.2. FTIR and DSC Analysis

**Figure 2:**
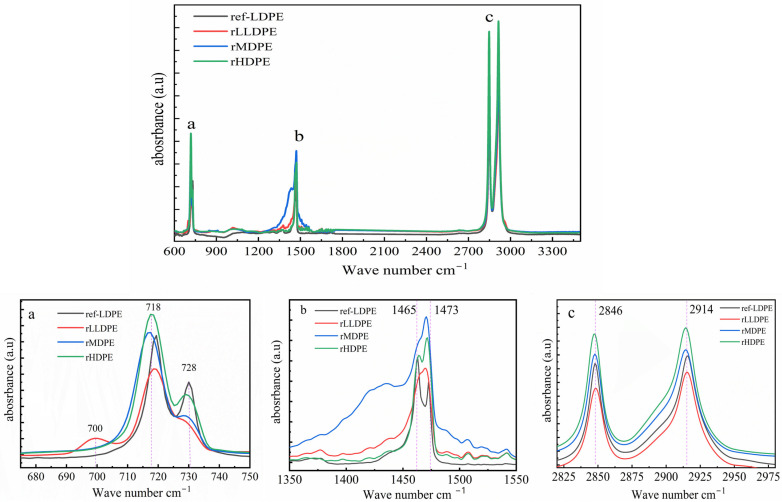
FTIR spectra of recycled PE waste plastics: methylene C–H rocking deformation in (CH_2_)_n_ (**a**), methylene C–H-bending deformation (**b**), and C–H asymmetric and symmetric stretching vibrations (**c**).

Figure 2a–c show the spectra of the three recycled PE polymeric plastics with characteristic regions separated into three parts of 675–750 cm^−1^ (a), 1350–1550 cm^−1^ (b), and 2825–2975 cm^−1^ (c), representing methylene (CH_2-_)-rocking deformation, C–H-bending deformation of CH_2_-, and C–H asymmetric and symmetric stretching of CH_2_-, respectively [51]. As shown in Figure 2, all three recycled PE plastics exhibited similar strong C–H asymmetric and symmetric stretching vibrations at 2914 and 2846 cm^−1^, respectively (c). Moreover, they depicted similar FTIR band regions at 718 and 728 cm^−1^ for methylene C–H rocking deformation in (CH_2_)_n_ (a) and methylene C–H-bending deformation at 1465 and 1473 cm^−1^ (b) compared with LDPE as a reference. The CH_2_ rocking peak in rHDPE split the peak near its shoulder at 718 and 728 cm^−1^ (a), whereas the split is not clear for rMDPE, indicating its lower crystallinity than rHDPE. However, rLLDPE displayed a broad peak in the medium-absorption region of 700 cm^−1^, which can be ascribed to the characteristic band region of the C–H plane bending in 1-hexene copolymerized with ethene [52]. The PE of higher crystallinity resulted in a higher splitting after a larger absorption, implying that the recycled polymeric plastic was properly sorted without other contaminants. Although the intensity and extent of splitting compared with the LDPE reference are different, this can be ascribed to the thermal reprocessing, which can reduce the regularity of the polymeric chains and affect the crystal structure [52]. Additionally, the absence of a carbonyl peak at 1725 cm^−1^ indicates the absence of oxidation of the recycled matrix during and/or degradation [51], except residual impurities. 

We used DSC to characterize recycled PE waste fractions to accurately predict whether their properties have remained intact after recycling. The evaluation was based on the fraction of crystallinity and melting characteristics, which we then compared with the literature to reasonably identify the differences between the PEs used.

**Figure 3:**
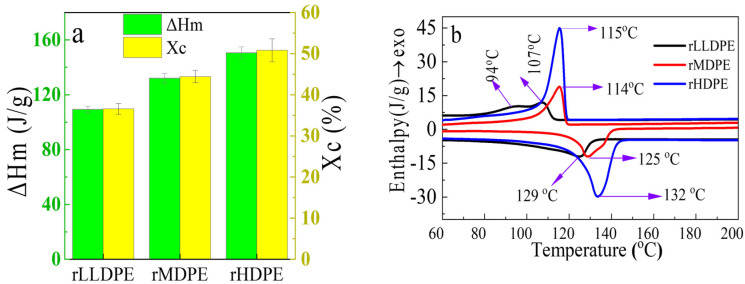
X_c_ and enthalpy of melting (**a**) and DSC thermograms of endothermic and exothermic heating cycle (**b**).

Figure 3a,b shows the DSC data of rLLDPE, rMDPE, and rHDPE, including their X_C_ and ΔH_m_. The T_m_ and X_c_ for the recycled PEs differ, as shown in Figure 3b. Recycled LLDPE exhibits the lowest Xc (a), with a broad, small, and less sharp melting peak (b), which is related to its large amorphous content. In contrast, both rMDPE and rHDPE maintained sharp crystallinity and melting properties, with rHDPE having a larger Xc value than rMDPE, which is consistent with the reported literature values for virgin PE with minor differences. For instance, rLLDPE was reported to have a weak crystallization peak at approximately 95 °C, followed by a sharp T_c_ at 109 °C, and a sharp T_m_ at approximately 123 °C. Similarly, virgin HDPE and MDPE were reported to have ΔH_m_ values of 152.89.7 and 144.3 J/g, respectively. [48]. Furthermore, a recent investigation on the DSC characteristics of four different neat PE polymers showed X_c_ values of 36.65%, 39.52%, 48.35%, and 51.28% for LDPE, LLDPE, MDPE, and HDPE, respectively [53,54]. These results are similar to our study findings, in which the Xc values were found to be 39.54%, 46.64%, and 51.08% for rLLDPE, rMDPE, and rHDPE, respectively. The slight variations are ascribed to the thermal processability affecting the crystallization of the polymer, with similar retained properties as their virgin counterparts. These results imply that thermomechanical recycling can retain properties like those of the respective virgin polymers and can be used as a secondary resource.

### 3.3. Contents of Metal Ions and Polycyclic Aromatic Hydrocarbons in the Recycled PEs

As shown in Table 3, the highest Ti, Ba, Cl, Zn, Ca, and Fe contents were detected in rLLDPE and rHDPE. However, in rMDPE, only Ba and Fe were detected in relatively large amounts. Such residual impurities can be attributed to the prehistoric production of their respective neat polymers, including residual catalysts of Neiglar Natta or metallocenes, inorganic pigments (TiO_2_, ZnO, and Fe_2_O_3_), flame retardants (Sb_2_O_3_ and brominated organics), and stabilizers of Ba, Sn, and Zn [12,46]. However, rMDPE was less contaminated, owing to its white opaque color. The content of heavy metals, such as Cd, should be less than 100 ppm (mg/kg) and that of Pd, Hg, and Cr (VI) should be less than 1000 ppm (mg/kg) [46]. The detected amounts below these thresholds indicate that the recovered PE can be used as a secondary resource for WPC formulations when properly sorted from the waste fractions. For others residual elements their threshold amounts are not available (n.a) implying their less toxicity for PE waste valorizations. Nevertheless, improved sorting and representative sampling should be performed continuously over an extended period.

**Figure 4:**
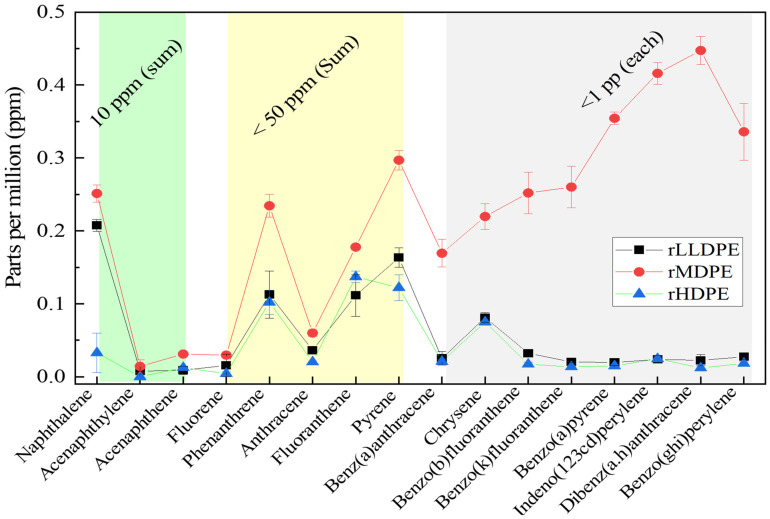
Contents of PAHs contents in the recycled rLLDPE, rMDPE, and rHDPE.

PAHs are hydrocarbons composed of multiple aromatic rings, which are recognized for their toxicity, carcinogenicity, and mutagenicity. Common PAHs and their detected amounts, including their threshold quantities, are shown in Figure 4. The combined total amounts of naphthalene, acenaphthylene, acenaphthene, and fluorene should be below 10 mg/kg. Similarly, the amounts of residual phenanthrene, anthracene, fluoranthene, and pyrene molecules must remain below 50 mg/kg (ppm), according to well-established standards for protection of the PAHs [45]. Benzo(a)anthracene, chrysene, benzo(b)fluoranthene, benzo(k)fluoranthene, benzo(a)pyrene, indeno(1,2,3-cd) pyrene, dibenzo(a, h)anthracene, and benzo(ghi)perylene concentrations did not exceed 1 mg/kg. The rMDPE has relatively high concentrations of polycyclic arenes compared with both rHDPE and rMDPE. The sources of theses PAHs could be residual impurity traces coming from their corresponding monomer and/or polymer productions, catalyst residuals and different impurities associated with additives during the productions. However, the PAHs levels detected in rLLDPE, rMDPE, and rHDPE complied with these requirements, making them suitable for valorization as secondary resources for the WPC core-matrix polymers. However, the evaluation requires continuous testing.

### 3.4. Mechanical Properties of the WPCs

The mechanical properties of the formulated WPCs, including the TS, TM, FS, FM, and UIS, are listed in Table 4 comprising the corresponding unfilled polymer matrix as a baseline for comparison. The subsequent discussion covers each of these key property variations with bamboo fibrous particles loading varied from 40% to 60%, complemented by the graphs for quick interpretation. The numbers in brackets represent the standard deviation of the ten specimens tested from two replicates for all formulations. In the UIS column, the letters “ub,” “pb,” and “b” were used to indicate unbroken, partially broken, and broken specimens, respectively.

#### 3.4.1. Tensile Strength and Tensile Modulus

**Figure 5:**
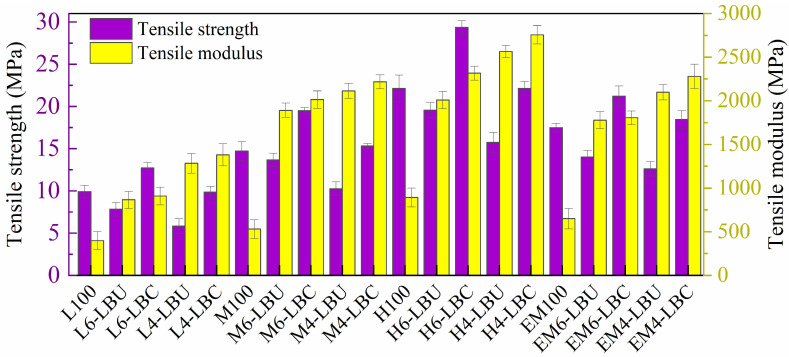
Tensile strength and tensile modulus of the WPCs.

As shown in Figure 5, WPCs formulated from the lower contents of the bamboo particles (40%) showed larger TS than those with 60%. This indicates a decreasing trend in TS with increasing bamboo particle loading, whereas the TM (stiffness) increases. Adding bamboo particles to a polymer matrix significantly enhanced the stiffness of both the coupled and uncoupled composites. The TM of both coupled and uncoupled WPCs with 40% bamboo particles, L_6_–LBU, L_6_–LBC, M_6_–LBU, M_6_–LBC, H_6_–LBU, H_6_–LBC, EM_6_–LBU, and EM_6_–LBC, were improved significantly with increasing ratios of 2.2, 2.3, 3.6, 3.8, 2.3, 2.6, 2.7, and 2.8, respectively, compared with that of unreinforced rLLDPE, rMDPE, rHDPE, and rEM. Furthermore, increasing the particle reinforcement to 60% from 30% increased the stiffness of the composites. Thus, the TM of 60% bamboo particles of both coupled and uncoupled composites, i.e., L_4_–LBU, L_4_–LBC, M_4_–LBU, M_4_–LBC, H_4_–LBU, H_4_–LBC, EM_4_–LBU, and EM_4_–LBC, increased by the ratios of 3.2, 8, 4, 4.2, 2.8, 3.1, 3.2, and 3.5, respectively, compared with the respective reinforced matrices. This depicts the pronounced effect of bamboo particles on increasing the stiffness of the composite compared with the coupling agents, which increased the TM to a maximum of 15%. Unlike TM, TS did not increase proportionally with the bamboo particle content in the composites without coupling agents because of fiber agglomerations that hinder the polymer chain encapsulation and weak adhesion. Nevertheless, for 40% bamboo particles of coupled WPCs, i.e., L_6_–LBC, M_6_–LBC, H_6_–LBC, EM_6_–LBC, the TS significantly increased by 27.8%, 32.5%, 32.6%, and 55.7%, respectively, compared with unreinforced polymeric matrices rLLDPE, MDPE, rHDPE, and EM. 40% bamboo particles of coupled WPCs also demonstrated 61.78%, 42.72%, 34%, and 51.3% increases compared with their respective uncoupled L_6_–LBU, H_6_–LBU, EM_6_–LBU composites, respectively. Similarly, the TS of coupled WPCs with 60% bamboo particles, i.e., L_4_–LBC, M_4_–LBC, H_4_–LBC, and EM_4_–LBC, showed significant increases of 68%, 49.3%, 46.1%, and 46%, respectively, compared with their respective uncoupled composites L_4_–LBU, M_4_–LBU, H_4_–LBU, and EM_4_–LBU. This trend shows that the MAPP compatibilizer enhances the interface compatibility, whereas composites without a coupling agent exhibit weak interphase compatibility, reducing the TS. This work achieved better results than WPCs produced from smaller particles and flour. In particular, the reported TS was 9.5–23.2 MPa for 30–50% dispersed phase, 3% MAPP (Epolene G-3015), and maleic-anhydride-grafted PE (MAPE). This improvement can be ascribed to the wider distribution of LLB particle reinforcement, higher cellulose content, or probably residual impurities that may enhance interactions, and higher grafted portions of MA in MAPP [55,56,57].

WPCs formulated by the melt-blending of equal amounts of rLLDPE, rHDPE, and rMDPE demonstrated better TS performance compared with both coupled and uncoupled rMDPE composites and 60% filled rHDPE-based composites. The TS of the EM-based composites EM_6_–LBU, EM_6_–LBC, EM_4_–LBU, and EM_4_–LBC greatly increased by 78.7%, 62.2%, 116%, and 87.11%, respectively, compared with the rLLDPE-based composites L_6_–LBU, L_6_–LBC, L_4_–LBU, and L_4_–LBC. These values are larger than the amount by which both TS and TM of corresponding rMDPE and rHDPE based composites were reduced compared to the respective rEM-based composites. In some cases, similar values are noted, particularly for the rMDPE composites. These findings indicate that melt-blending of highly polluting and weak rLLDPE with stronger rMDPE and rHDPE resulted in a composite with unique characteristics, despite their inherent immiscibility. The intrinsic immiscibility is further enhanced by MAPP, which can be ascribed to its hydrophobic part interacting with nonpolar EM chain molecules and polar-ending groups of MA effectively combining with hydrophilic bamboo reinforcements through hydrogen bonding or ester bonds [58]. These phenomena were indicated by significant changes in the FTIR spectra of both the LLB-reinforced EM-based coupled and uncoupled composites. Furthermore, the mechanical interlocking of a wider particle distribution and molecular entanglement in mixed PE poly blends are considered to inhibit the mechanical failure at the weakest point of rLLDPE. Similarly, high-temperature compounding and high-pressure compaction can also reduce the viscosity and enhance the matrix flow to encapsulate bamboo reinforcement.

#### 3.4.2. Bending Strength (FS) and Modulus of Rupture (FM)

**Figure 6:**
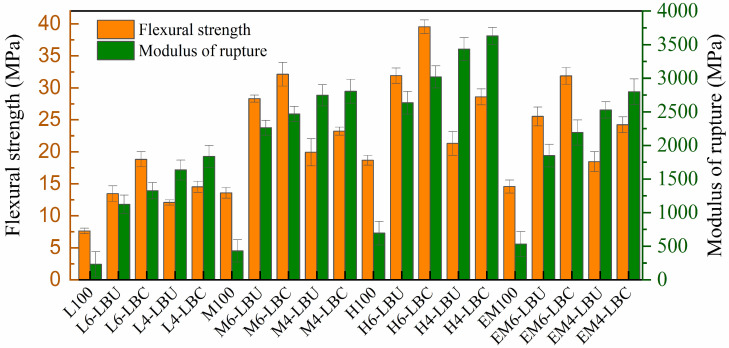
The flexural strength of WPCS.

The FS and FM results are shown in Figure 6. The FS of the formulated composites increased for both coupled and uncoupled composites compared with that of the core polymer, unlike the TS. Bamboo-particle reinforcement improved the low FS of all unreinforced matrices. However, the FS decreased slightly with increasing bamboo particle contents. Similarly, the FM was improved compared with that of the unreinforced composites, exhibiting increasing trends with increasing LLB content. The FM of both coupled and uncoupled WPCs with 40% LLB particles, i.e., L_6_–LBU, L_6_–LBC, M_6_–LBU, M_6_–LBC, H_6_–LBU, H_6_–LBC, EM_6_–LBU, and EM_6_–LBC, were significantly improved by the ratios of 4.3, 4.9, 5.3, 5.8, 3.8, 4.4, 3.6, and 4.1, respectively, compared with unreinforced rLLDPE, rMDPE, rHDPE, and rEM. Similarly, FM uncoupled and coupled WPCs with 60% LLB particle, i.e., L_4_–LBU, L_4_–LBC, M_4_–LBU, M_4_–LBC, H_4_–LBU, H_4_–LBC, EM_4_–LBU, and EM_4_–LBC, resulted in increasing trends of 7.1, 7.9, 6.4, 6.5, 5, 5.2, 4.7, and 5.3 ratios, respectively, compared with their unreinforced matrices of rLLDPE, rMDPE, rHDPE, and rEM. However, FM only marginally increased with MAPP inclusion at both 40% and 60% LLB particles.

The FS of both uncoupled and coupled WPCs with 40% LLB particles, i.e., L_6_–LBU, L_6_–LBC, M_6_–LBU, M_6_–LBC, H_6_–LBU, H_6_–LBC, EM_6_–LBU, and EM_6_–LBC, exhibited increasing trends by the ratio of 1.8, 2.5, 2, 3.4, 1.7, 2.1, 1.8, and 2.2, respectively, compared with the unreinforced matrices of rLLDPE, rMDPE, rHDPE, and EM. Similarly, the FS of uncoupled and coupled WPCs with 60% LLB particles, L_4_–LBU, L_4_–LBC, M_4_–LBU, M_4_–LBC, H_4_–LBU, H_4_–LBC, EM_4_–LBU, and EM_4_–LBC, increased by 1.58, 1.9, 1.5, 1.7, 1.2, 1.5, 1.3, and 1.7 compared with those of respective unreinforced rLLDPE, rMDPE, rHDPE, and rEM. Additionally, the FS of coupled WPCs with the same LLB particle content (40% and 60%) demonstrated significantly increasing trends with MAPP inclusion compared with uncoupled samples. Furthermore, the FS of the EM-based composites EM_6_–LBU, EM_6_–LBC, EM_4_–LBU, and EM_4_–LBC increased by 89.73%, 69.23%, 52.7%, and 66.75%, respectively, compared with their respective rLLDPE-based formulations. Similarly, the FM of the EM-based composites increased by 90%, 97%, 54%, and 52% compared with L_6_–LBU, L_6_–LBC, L_4_–LBU, and L_4_–LBC, respectively. In conventional WPC applications for decking and wall cladding with 40–60% wood fibers or polymers, the minimum recommended FS and FM are 20 and 1200 MPa, respectively [59]. For nonstructural applications, such as ceilings, sidings, and modular kitchen interiors, the minimum TS and FS are 10 and 15 MPa, respectively, according to the ISO 178-1 [48], ASTM D6109 [60], and ASTM D7032 [61] standards. The formulated coupled WPCs satisfied these requirements, similar to EM-based composites. Although most PE-based composites use MAPE as the coupling agent, our results are consistent with those of other studies using various coupling agents. These include functionalized polyolefins, maleic-anhydride-grafted LLDPE (LLDPE-*g*-MA), acrylic-acid-grafted HDPE (HDPE-g-AA), MAPP, and maleic-anhydride-grafted styrene-ethylene-butylene-styrene, as extensively reviewed by Kaseem et al. [17]. They found that WPCs containing MAPP and HDPE-AA exhibited better properties, except that the maximum impact strength (IS) achieved when LLDPE-g-MA was used as a coupling agent, which can be ascribed to the intrinsically high IS of its main block polymer, LLDPE. It is also noted that a MAPP with a higher MA content was used in this study to evaluate its performance in the PE blend matrix, as many commercial WPCs for building materials use MAPP due to its lower cost compared to PE-g-MA. Additionally, MAPP is more thermally stable and widely used in commercial applications, making it ideal for comparison.

#### 3.4.3. Impact Strength (IS)

**Figure 7:**
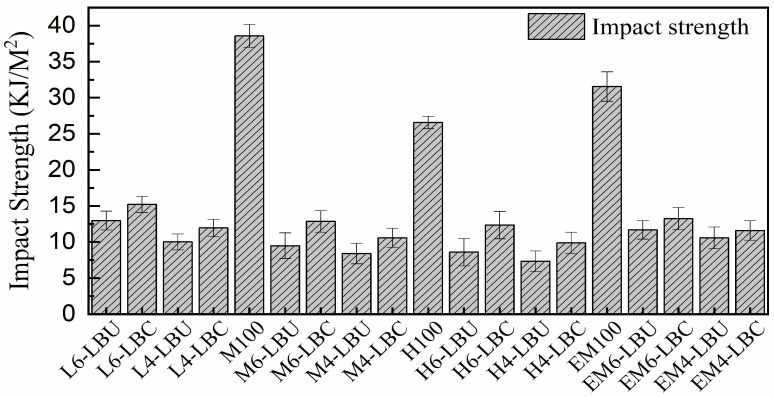
The impact properties of WPCs.

In the IS evaluation, the rLLDPE matrix remained unbroken with the ISO pendulum used, whereas rMDPE and rEM were partially broken, and rHDPE was fully broken. As indicated in Figure 7, the rLLDPE results were not included in the graph, and the IS was evaluated based on the other WPCs. Accordingly, the IS of the WPCs was in the range of 7.34 kJ/m^2^ (H_4_–LBU) to 18.2 kJ/m^2^ (L_6_–LBC) without considering the polymeric matrix. Increasing the LLB content from 40% to 60% reduced the IS. Additionally, formulations without MAPP exhibited a low IS, which is likely attributable to the weak interfacial compatibility. However, the IS of both rMDPE- and rHDPE-based WPCs improved when melt-blended with MAPP and rLLDPE owing to their increased ductility and compatibility with the blend matrix. The IS of the WPCs with short fibrous particles was significantly affected by the rigidity of the natural fibers, restricting the polymer chain mobility, reducing the energy absorption during fracture, and breaking the composite [62,63]. Except for H_4_–LBU, the WPCs satisfied the minimum requirements for a wide range of practical applications [59]. In particular, an IS higher than 8 kJ/m^2^ is recommended for structural WPCs for applications to outdoor decking, according to ISO 179-2 [49] test compliance. Non-load-bearing applications have less strict requirements, requiring WPCs that are stiff and not easily ruptured, thereby meeting minimum standards, similar to plastic lumber, with an FS of 6.9 MPa and FM of 340 MPa [64]. MAPP inclusion improved the IS of the WPCs by enhancing the LLB particle dispersion and interphase bonding, increasing the energy absorption and resistance to breakage [65].

The IS of the EM-based composites EM_6_–LBU, EM_6_–LBC, EM_4_–LBU, and EM_4_–LBC increased by 36.8%, 31.4%, 44%, and 34% compared with their unblended rHDPE-based matrices of H_6_–LBU, H_6_–LBC, H_4_–LBU, and H_4_–LBC, respectively, and increased by 23%, 18%, 16%, and 15% compared with the respective unblended rMDPE-based composites of M_6_–LBU, M_6_–LBC, M_4_–LBU, and M_4_–LBC, respectively. Furthermore, the IS of EM_6_–LBC and EM_4_–LBC increased by 71% and 73% compared with that of M_6_–LBU and M_4_–LBU, respectively, and by 89% and 98% compared with that of uncoupled rHDPE matrices H_6_–LBU and H_4_–LBU, respectively.

The improved IS in all coupled WPCs compared with that in the uncoupled WPCs indicates the improved chemical interactions, rather than the physical interlocking. In WPCs without coupling agents, the IS decreased owing to the weak interfacial bonding between the filler and polymer matrix, facilitating microcrack initiation and reducing the energy required for crack propagation. The coupling agents improved IS by reducing the viscosity, enhancing the matrix flow, ensuring uniform fiber distribution, and improving the interfacial adhesion between the LLB particles and matrix through chemical reactions [7]. The results and trends align with those of Sommerhuber et al. [7], showing that the addition of wood fibers to the WPCs formulated from neat and recycled HDPE with 3% MAPE and Norway spruce fibers leads to decreasing IS. Specifically, the IS values were in the range of 7.6–8.4 kJ/m^2^ at 30% wood-fiber content and 5.4–8.0 kJ/m^2^ at 60% wood-fiber content, indicating improvement in IS with MAPE inclusion. Similarly, Migneault et al. [66], Poletto et al. [67], and Khamtree et al. [62] noted that using MAPP, PEMA, and styrene-maleic anhydride significantly improved the IS. Compared with these studies, enhanced toughness was observed in the EM-based WPCs formulated in this study, which could be attributed to both the coupling efficiency of MAPP and intrinsic toughness of rLLDPE.

### 3.5. SEM-Microscopic Characterization

**Figure 8:**
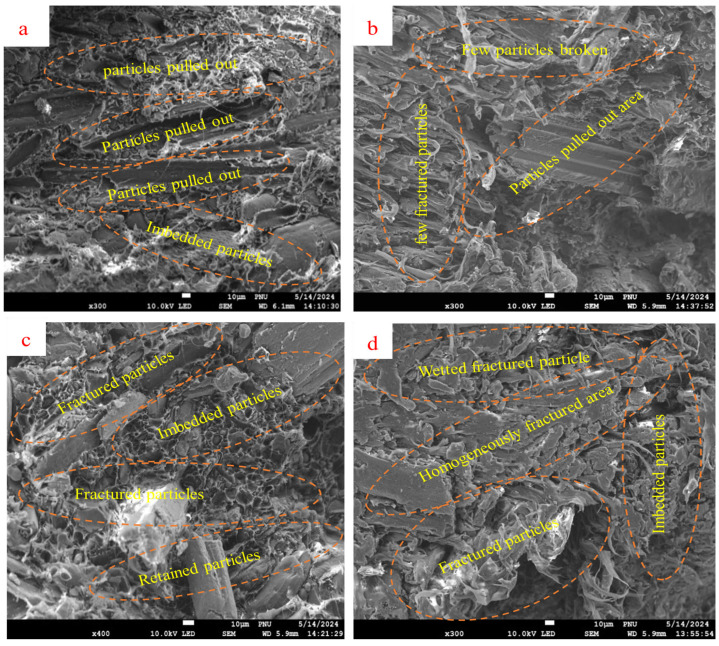
SEM morphology of EM-based WPC samples of both uncoupled composites (EM–LBU) at two site points (**a**,**b**) and coupled composites (EM–LBC) at two different site points (**c**,**d**).

The SEM morphologies of the WPC surfaces formulated from equal compositions of the representative polymer blends (EM) obtained by the fractured IS test are shown in Figure 7. The analysis included uncoupled WPCs (EM_6_–LBU) (Figure 8a,b) and coupled WPCs (EM_6_–SLBC) (Figure 8c,d), which were each examined twice from two replicates of the same formulation. As shown in Figure 8a,b, several smaller particles were removed from the matrix during the IS testing, leaving large particles pulled out from the areas, smaller holes, and loosely embedded particles. This can be ascribed to the incompatibility, resulting in the weak interactions and reduced mechanical properties of the composite. Conversely, coupled EM_5_–LBC (Figure 8c,d) demonstrated good dispersion of the bamboo fibrous particles in the polymer matrix without visible agglomerates and fiber pull-out areas. Furthermore, several particles were homogeneously embedded within the matrix (Figure 8c). Similarly, the morphology was uniformly distributed across the matrix composites, covering well-oriented surface layers without particle-matrix deformation (Figure 8d). The absence of clear voids or particle agglomerates indicates the reduced fiber pull-out areas and improved interfacial bonding between the particles and polymer matrix by the compatibilizer. This leads to improved mechanical properties compared with their uncoupled counterparts. The absence of large voids and gaps, including fractured fiber surfaces, has also been reported to confirm the presence of a strong fiber–polymer interphase and coupling efficiency [7,12,68]. 

### 3.6. FTIR Characterization of WPCs

**Figure 9:**
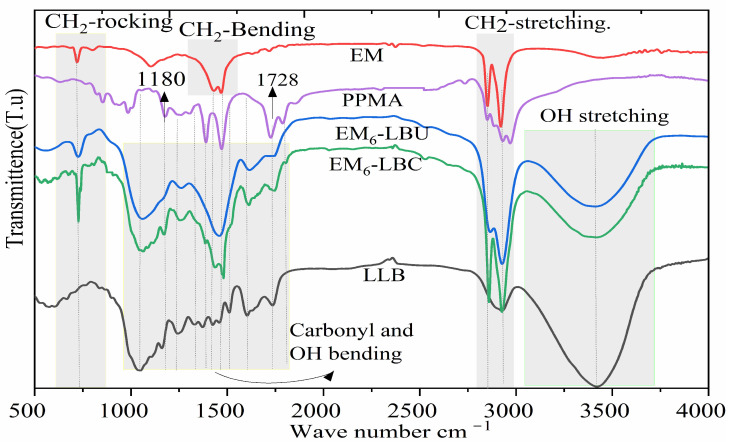
FTIR spectra of WPCs based on the equal blend ratio (EM) of (LLDPE, MDPE, and HDPE).

The representative FTIR spectra of the coupled and uncoupled EM-based WPCs and LLB particles are shown in Figure 9, with the spectra shifted vertically for clarity. The fingerprint regions of most lignocellulosic fibers at 600–1600 cm^−1^ represent major constituents, such as cellulose, hemicelluloses, and lignin molecular vibrations [69]. Similarly, the broad spectral region within 3250–3500 cm^−1^ represents the hydroxyl (-OH)-stretching vibration of mostly cellulose and hemicellulose. The other important regions from 1060 to 1030 cm^−1^ were assigned to C–O stretching of mostly cellulose, and the region from 1750 to 1700 cm^−1^ represents carbonyl region carboxylic acid and ester groups [29].

As shown in Figure 9, MAPP reduces the –OH functionalities of the LLB particles, as depicted by the weakening of the OH stretching at approximately 3430 cm^−1^ and C–O lignin stretching at approximately 1050 cm^−1^ [27] with changes in the peak intensity length, denoting chemical changes [70]. Additionally, several typical vibration peaks of cellulose, hemicellulose, and lignin biomass in the complex regions of 1152–1755 cm^−1^ were affected compared with those of the coupled composites. These included OH– in-plane bending and lignin C–H bending in CH_3_ at 1454 cm^−1^, lignin aromatic skeleton vibrations at 1508 cm^−1^, and C–H bending at 1462 cm^−1^. Moreover, hemicellulose COO stretching, lignin C–O stretching, hemicellulose (C–O stretching in carboxylic acid), and lignin (C–C, C–O, C=O) stretching are noted at 1380, 1264, 1240, and 1226 cm^−1^, respectively [62,63]. Compared with LLB, some of these absorption peaks disappeared and/or shifted to lower or weaker intensities in the coupled WPCs. The anhydride carbonyl group of MA in PP (1180 cm^−1^) was detected in the coupled composites. Although the LLB particles have an ester band region at approximately 1728 cm^−1^ [70], compared with the EM_6_–LBC composites, this band region was clearly observed in EM_6_–LBC. Similarly, the CH_2_-stretching vibration peaks at 2920 and 2852 cm^−1^ and CH_2_-bending vibrations were more intense in the EM_6_–LBC systems than in EM_6_–LBCU, implying improved interactions with the hydrophobic blend chains. These phenomena indicate synergetic interphase modification with MAPP and polymer blending, resulting in improved mechanical properties, as observed during mechanical property characterization. A relatively weak peak around 1093 cm^−1^ and 870 cm^−1^ in the EM most likely represent the out-of-plane and in-plane bending vibrational modes of CO_3_^2−^ in CaCO_3_, confirming the presence of this mineral filler in the recycled PE waste [71].

### 3.7. Thermogravimetric Analysis of WPCs

**Figure 10:**
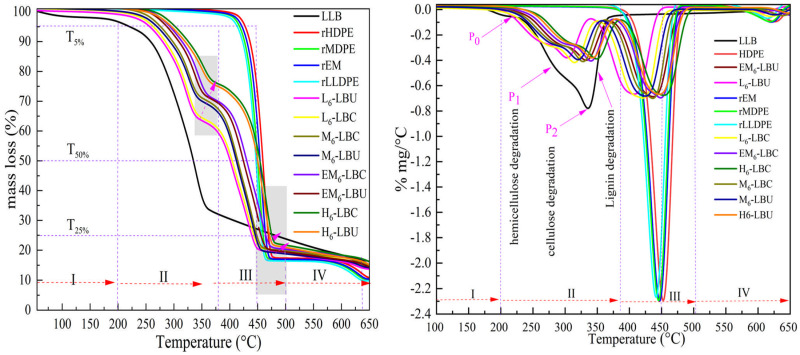
The TGA (**left**) and DTGA (**right**) curves of the WPCs and its polymer matrices.

The initial mass decrease (ca. 2.5%) of LLB may be due to the moisture absorption, though LLB well dried before grinding and sealing to prepare WPCs. As shown in Figure 10 and Table 5, however, the temperature at 5% mass loss (T_5%_) for LLB is 206 °C, which is higher than previously reported values for other bamboo fibers [72]. This could be attributed to the high amounts of cellulose and stable lignin, as inferred from the chemical bamboo characterizations. The first shoulder peak (P_0_) appeared in region II (206–236 °C) with a weight loss of 9.65%. At 236–372 °C (including P_1_ and P_2_), a weight loss of 68.5% was noted owing to cellulose and lignin decomposition, especially at P_2_ at approximately 365 °C. In stage III (365–600 °C), complete LLB decomposition occurred with a mass loss of 18.54% due to the oxidative decomposition of carbonized residue and volatilization of anhydrous sugars, leaving a residual char of 13.07%. Similarly, Chakkour et al. [72] observed two major decomposition peaks (P_1_ and P_2_), with P_2_ being the most prominent. In our case, the overlap of hemicellulose and cellulose at high temperatures for P_1_ (315 °C) and a larger mass loss rate at P_2_ are likely attributable to the low hemicellulose and high cellulose contents of the LLBs.

Incorporating LLB fibrous particles into WPCs changed the thermal degradation into multiple stages, decreasing it below that of the unreinforced matrix polymers, particularly up to region II. In region I, the degradation below 200 °C includes volatilization of residual water and extractives and degradation of amorphous hemicellulose of LLB incorporated into the WPC matrix. The major degradation between regions II and III is ascribed to the decomposition of the cellulose, hemicellulose, and lignin fibrous bamboo particles, which mostly occurred below the decomposition of the polymeric matrix. Hemicellulose that degraded earlier continued to degrade in the temperature range of 270–360 °C in region II, along with the active pyrolysis of the remaining cellulose (α-cellulose). Lignin is a thermally stable and highly networked phenolic molecule that decomposes over a wide range and contributes to char formation at 510 °C and higher [72]. Similarly, the peak intensity in region III was greater than that in region II because of the significant degradation of the WPC polymer matrix in region III. Region IV is likely ascribed to the complete decomposition and evolution of the pyrolysis gas products and their final decomposition to ash.

The WPCs containing MAPP exhibited higher thermal stability than the uncoupled composites because of the improved interfacial force and encapsulation of the fibers within the polymeric matrix. Similarly, it shifted T_5%_ and T_max_ at the end of region III to higher values. Specifically, in region III between T_50%_ and T_15%_, the T_max_ of the WPCs of EM and rHDPE with MAPP exceeded the degradation temperature of their corresponding matrix with increased residue [72]. It was reported that MAPP improved the interphase and catalytically carbonized [73] and promoted the release of high-carbon char from lignin, which acted as a thermal barrier during its wide thermal degradation range because of its stable aromatic phenyl groups [74]. This phenomenon enhanced the thermal stability of the coupled melt-blended WPCs of the polymers in region III compared with the unreinforced polymeric matrix and coupled and uncoupled WPCs based on rLDPE and MDPE. Additionally, EM-based composites benefit from the high thermal stability of the rHDPE matrix, improved fiber encapsulation, and efficient dispersion due to MAPP. The T_max_ of the EM matrix (477.9 °C) in region III shifted to 486.8 °C in EM6–LBC, realizing complete oxidation at temperatures above 500 °C. However, beyond 500 °C at the end of region III, the carbonization effects of the LLB decreased, leading to complete WPC degradation. Free radicals from coupling agents and polymer matrix degradation catalyzed composite pyrolysis and increased reactivity, resulting in minimal differences in the remaining mass at 650 °C compared with LLB [73,75]. However, with the rising temperature the detectable decomposition ranges is revealed around 600 °C with 6.24% mass loss. This second phase could originate from the slow pyrolysis of inorganic mineral fillers such as CaCO_3_ as high amounts of Ca were found in our previous determination of metal additives.

WPCs with neat and recycled HDPE using pine and bagasse fibers, coupled with PEMA and carboxylated polyethylene, have T_max_ values of approximately 470 °C. A slight increase in T_max_ of over 19 °C in our study is likely attributable to the residual mineral fillers and improved MAPP compatibility. Similarly, WPCs from rHDPE and commercial wood fibers (poplar, Douglas fir, black locust, white oak, and ponderosa pine) started to degrade at approximately 240 °C, whereas the ponderosa pine-based WPCs started to degrade at 259 °C [76]. All PE-based recycled WPCs (both coupled and uncoupled), including the EM-based composites, performed well, except for the rLLDPE-based composites. In particular, the obtained thermogravimetric degradation characteristics are similar to and/or better than those previously reported and commercial WPCs made from neat PE [17,52].

## 4. Conclusions

This study developed new WPCs by combining rPE waste fractions (rLLDPE, rMDPE, rHDPE) with indigenous Ethiopian bamboo particles (*Oxytenanthera abyssinica*, i.e., “LLB”) as reinforcements with key findings outlined below. In this study, WPC formulations consist of either of the three different recycled polyethylene (rLLDPE, rMDPE, or rHDPE), or their blend with equal composition (33/33/33 by wt.%) as the matrix and short fibrous LLB particles (200–600 µm) as the dispersed phase, without any solvent-based surface treatment.

Indigenous Ethiopian bamboo fibrous particles contain high levels of cellulose, lignin, and hemicellulose, making it a potential substitute to conventional wood fibers in WPC formulations. The residual elements (Ti, Ba, Cl, Zn, Pd, Hg, Br, and Cr VI) in the PE waste fractions and PAHs were below the threshold limits and remained intact during recycling, as confirmed by FTIR, DSC, and TGA, ensuring their suitability as a secondary resource for WPC formulation instead of waste.

In situ reactive compounding with MAPP greatly enhanced the interfacial strength compared with the unfilled polymer matrices through thermally induced chemical coupling and effective dispersion of bamboo particles for mechanical interlocking. These result in enhanced mechanical properties, improved thermal degradation, reduced −OH band intensities, changes in carbonyl regions, and removal of some of the characteristic LLB band regions, including reduced fiber pull-outs in the coupled composites.

The ultimate TS and FS decreased with an increase in LLB loading from 40% to 60%. Improved mechanical properties were observed at 40% loading due to the improved melt-flow and enhanced matrix encapsulation. However, at 60% loading, significant mechanical properties were maintained with the use of MAPP. Notably, the bending strength of the unreinforced matrix and its FM markedly increased for all formulations. The TM and FM increased with the LLB loading, with minor variations observed with the coupling agents. Moreover, WPCs formulated from coupled mixed blends of rLLDPE, rMDPE, and rHDPE showed improved mechanical properties by MAPP compatibilization and melt-blending of rLLDPE with rMDPE and rHDPE. The IS of the EM-based composites increased by 31.4% to 44%, compared with their unblended rHDPE-based matrices. Furthermore, the IS of EM_6_–LBC and EM_4_–LBC increased by 71% and 73% compared with that of M_6_–LBU and M_4_–LBU, respectively, and by 89% and 98% compared with that of uncoupled rHDPE matrices H_6_–LBU and H_4_–LBU, respectively. Similarly, thermal stability of the WPCs produced from EM-based composites with the coupling agent inclusion benefit from the high thermal stability of the rHDPE matrix, compared with the unreinforced polymeric matrix and WPCs based on both rLLDPE and rMDPE matrix. The MAPP improved fiber encapsulation and efficient dispersion within the matrix polymers, which highly improved the thermal degradation of the LLB particles.

These WPCs offer practical alternatives to polyolefin-based composites with wood fiber or polymer contents of 40–60%, satisfying the standards for FS, FM, and TS, respectively. Formulated WPCs could be suitable for furniture, wall cladding, ceiling boards, interior designs, decking, and construction applications, such as insulation, with low biodegradation risk and deformation, with properties comparable to commercial products.

Future studies should focus on further enhancing the IS of the composites using dual-functional coupling agents with multiple monomers for broader property improvements. Exploring alternative production methods, such as extrusion and injection molding, is crucial for achieving better particle distribution within the polymer matrix. Moreover, benchmarking the PE waste fractions and identifying target applications can unlock the full potential of WPCs. Further research is also needed to understand the impact of recycled thermoplastics on the durability of WPCs and optimize the product properties.

## Figures and Tables

**Table 1:** Characteristics of the recycled PE polymers and MAPP.

Polymers	MFI (g/10 min)	Density (g/cm^3^)
rLLDPE	1.98	0.92
rMDPE	1.25	0.93
rHDPE	0.2	0.96
MAPP	21	0.934

**Table 2:** Formulations of WPCs samples.

Polmer Matrix Type	Polymer Matrix (%)	LLB Reinforcement (%)	Coupling Agents (MAPP)	Sample ID
**rLLDPE**	100	-	-	L_100_
	60	40	-	L_6_–LBU
	58.5	38.5	3	L_6_–LBC
	40	60	-	L_4_–LBU
	38.5	58.5	3	L_4_–LBC
**rMDPE**	100	-	-	M_100_
	60	40	-	M_6_–LBU
	58.5	40	3	M_6_–LBC
	40	60	-	M_4_–LBU
	38.5	58.5	3	M_4_–LBC
**rHDPE**	100	-	-	H_100_
	60	40	-	H_6_–LBU
	58.5	38.5	3	H_6_–LBC
	40	60	-	H_4_–LBU
	38.5	58.5	3	H_4_–LBC
**rEM**	100	-	-	EM_100_
	60	40	-	EM_6_–LBU
	58.5	40	3	EM_6_–LBC
	40	60	-	EM_4_–LBU
	38.5	58.5	3	EM_4_–LBC

**Table 3:** Contents of main residual metal and non-metal elements in the recycled PE (rLLDPE, rMDPE, and rHDPE) with the standard deviation in the brackets.

Element	rLLDPE (ppm)	rMDPE (ppm)	rHDPE (ppm)	Threshold Amount (ppm)
Ti	314.8 (2.7)	26.9 (4.6)	805.9 (9.3)	n.a
Cl	735.8 (7.4)	36.4 (2.91)	305.2 (7.87)	n.a.
Fe	626.8 (2.4)	137.1 (5.8)	315.6 (2.4)	n.a
Ca	128.6 (9.6)	78 (0.23)	1056.3 (12.6)	n.a
Ba	1226.5 (56.3)	133.9 (2.17)	225.7 (9.2)	n.a
Cr	8.2 (0.68)	18.6 (0.72)	22.7 (0.78)	10^3^
Sn	12.2 (0.5)	12.1 (0.33)	7.5 (0.1)	n.a
Cd	4.4 (0.3)	3.8 (0.26)	6.3 (0.3)	10^2^
Pd	2.3 (0.1)	1.3 (0.8)	177.6 (0.2)	10^3^
Hg	2.3 (0.3)	3.8 (0.45)	1.8 (0.4)	10^3^
Zn	81.8 (4.5)	3.2 (0.1)	282.1 (3.5)	n.a
Cu	5.1 (0.7)	4.3 (0.4)	14.9 (0.8)	n.a
Mn	51.5 (2.3)	2.4 (0.79)	19.1(1.2)	n.a

**Table 4:** Mechanical properties of formulated WPCs.

Matrix	SID	TS (MPa)	TM (MPa)	FS (MPa)	FM (MPa)	UIS (KJ/m^2^)
LLDPE	L_100_	9.93 (1.73)	398.98 (53)	7.63 (0.45)	230.57 (88)	ub
	L_6_–LBU	7.85 (0.87)	866.60 (48)	13.45 (1.23)	1123.67 (37)	12.97 (1.3) (pb)
	L_6_–LBC	12.73 (0.2)	909.85 (52)	18.82 (1.18)	1324.65 (21)	15.21 (2.1) (pb)
	L_4_–LBU	5.84 (0.1)	1284.38 (63)	12.08 (0.41)	1634.68 (48.5)	10.01 (2.1)
	L_4_–LBC	9.86 (0.6)	1382.26 (77)	14.53 (0.87)	1835.6 (63.6)	11.95 (0.22)
MDPE	M_100_	14.72 (1.38)	530.75 (57)	13.58 (0.84)	428.94 (66)	38.56 (1.58) (pb)
	M_6_–LBU	13.67 (0.56)	1891.8 (32)	28.316 (0.55)	2263.4 (11.5)	9.49 (0.79)
	M_6_–LBC	19.51 (0.2)	2014.5 (50)	32.1 (1.86)	2467.2 (14.78)	12.86 (0.99)
	M_4_–LBU	10.27 (0.1)	2113.8 (38)	19.93 (2.12)	2745.8 (57.56)	8.41 (0.42)
	M_4_–LBC	15.33 (0.8)	2218.7 (30)	23.23 (0.162)	2804.8 (104.14)	10.58 (0.33)
HDPE	H_100_	22.15 (1.58)	892.85 (58)	18.6 (0.76)	693.58 (76)	26.56 (0.85)
	H_6_–LBU	19.57 (0.5)	2010.46 (50)	31.89 (1.2)	2634.6 (68.67)	8.59 (1.9)
	H_6_–LBC	29.37 (0.1)	2317.7 (30)	39.54 (2.32)	3019.5 (165.32)	12.35 (0.9)
	H_4_–LBU	15.75 (0.2)	2565.7 (22)	21.308 (2.12)	3433.2 (73.52)	7.34 (0.44)
	H_4_–LBC	22.15 (0.1)	2756.8 (55)	28.58 (3.15)	3628.6 (28.2)	9.89 (0.47)
EM	EM_100_	17.5 (0.5)	650.67 (68)	14.561.05)	530.87 (87)	31.56 (3.05) (pb)
	EM_6_–LBU	14.03 (0.7)	1778.85 (47)	25.52 (1.6)	1847.9 (69)	11.68 (0.27)
	EM_6_–LBC	21.23 (0.9)	1806.84 (27)	31.85 (1.3)	2191.5 (87)	13.24 (2.14)
	EM_4_–LBU	12.63 (0.6)	2099.36 (37)	18.45 (1.48)	2527.8 (20.6)	10.58 (0.25)
	EM_4_–LBC	18.45 (0.4)	2280.88 (90)	24.23 (1.44)	2798.5 (111.4)	11.60 (0.36)

**Table 5:** Thermographic data of polymer matrix and its WPCs.

Sample ID	T_5%_ (°C)	T_50%_ (°C)	T_25%_ (°C)	T_max_ (II) (°C)	Remaining Mass (%) at T_max_ (II)	T_max_ (III) (°C)	Remaining Mass (%) at T_max_ (II)	Remaining Mass at 650 °C
LLB	225.8	335.6	460.2	361	33. 10	-	23.13	16.7
rHDPE	427.9	458.9	469.76	-	-	480.9	17.8	10.8
rMDPE	417.2	451.6	459.3	-	-	474.8	17.4	10.2
rEM	421.6	453.6	464.6	-	-	477.9	17.6	10.9
rLLDPE	412.5	447.4	455.4	-	-	470.5	16.8	10.0
L_6_–LBU	265.5	408.5	442.6	341.8	65.1	456.7	20.8	13.7
L_6_–LBC	274.8	412.7	439.5	352.1	65.4	462.2	20.6	14.7
EM_6_–LBC	298.4	440.8	468.2	377.5	72.1	486.8	21.48	16.1
EM_6_–LBU	292.1	434.3	464.8	369.7	71.4	475.5	20.9	14.5
M_6_–LBC	283.8	423.5	464.8	363.1	70.4	473.8	21.2	15.1
M_6_–LBU	276.8	417.8	456.8	357.7	69.6	468.1	20.8	14.2
H_6_–LBC	309.8	462.9	485.8	387.5	75.6	496.9	21.9	15.1
H_6_–LBU	300.6	455.5	471.0	379.1	76.3	481.6	22.6	16.2

## Data Availability

All relevant data are presented and included in the article.
